# An atypical presentation of sporadic ovarian Burkitt’s lymphoma: case report and review of the literature

**DOI:** 10.1186/1757-2215-6-46

**Published:** 2013-07-04

**Authors:** Paola Bianchi, Francesco Torcia, Marta Vitali, Giuliana Cozza, Marco Matteoli, Valentina Giovanale

**Affiliations:** 1 Department of Gynecology-Obstetrics and Urological Sciences, “Sapienza” University of Rome, S. Andrea Hospital, Rome, Italy; 2 Department of Radiology “Sapienza”, University of Rome, S. Andrea Hospital, Rome, Italy

**Keywords:** Burkitt, Ovarian lymphoma, Hypoaesthesia, Oophorectomy, Chemotherapy

## Abstract

Primary non-Hodgkin’s lymphoma of the ovary is a rare occurrence. An ovarian involvement by non-Hodgkin lymphoma (NHL) may include one of the four subtypes of lymphoma: diffuse large B-cell lymphoma, Burkitt’s lymphoma (BL), lymphoblastic lymphoma or anaplastic large cell lymphoma. Burkitt’s lymphoma is a rare entity with a specific poorly differentiated pattern.

Most women experience an ovarian BL with abdominal pelvic pain, abnormal vaginal bleeding, bowel obstruction, urinary frequency, incontinence and abdominal mass. Sometimes these warning signs may be absent, causing a late and more difficult diagnosis.

Here we report a case of a primary ovarian Burkitt’s lymphoma with bilateral involvement in a 57 year old patient. She firstly presented neurological symptoms in the upper limbs and she was treated with surgery and combined chemotherapy. The diagnosis of malignant lymphoma was established after bilateral adnexectomy and histological study of the excised tissue.

## Case report

A 57-year-old pluriparous woman from Pakistan was admitted to the emergency ward due to a rapidly progressive weakness in her arms, bilateral hypoaesthesia of the 1st, 2nd and 3rd finger and wide-spread pain in her arms, shoulders and neck. Her medical history shows a TBC infection at the age of 17. MRI revealed volumetric increase of right C5-C6-C7 and left C6-C7 nerve roots with a mild contrast enhancement as idiopathic neuritis. Rachicentesis was negative. Serological HIV, HBV, HCV, neurotropic virus tests and autoantibodies were negative. The hematologic findings showed a low lymphocyte count and a slight increase in neutrophils.

**Figure 1:**
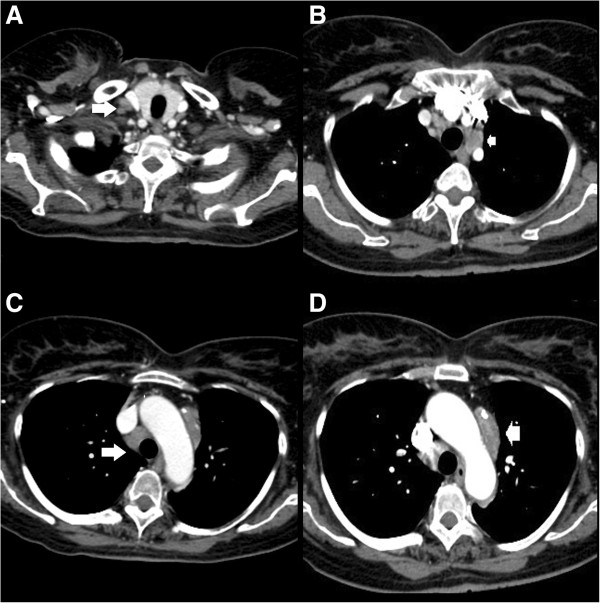
**CT scan of mediastinum-axial section.** CT scan of mediastinum shows diffuse lymphatic swelling (arrow) over right supraclavicular **(A)**, pre-vascular **(B-D)** and aortopulmonary **(C)** station with inhomogeneous enhancing after contrast media, suggestive for lymphoma.

A Computer Tomography (CT) scan revealed multiple enlarged lymph nodes involving the bilateral supraclavicular, left laterocervical, left and right paratracheal, prevascular, aortopulmonary, left and right hilar and subcarinal groups (Figure 1).

**Figure 2:**
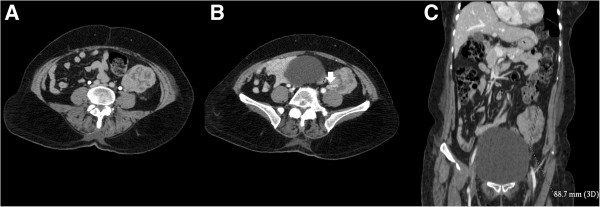
**Pelvic CT scan-axial and coronal sections.** CT scan of pelvis in arterial **(A)** and portal **(B-C)** phase, shows an inhomogeneous neoformation 65x51x88mm in correspondence of left ovary, with multiple necrotic areas within and a slow-enhancing after contrastum media administration. Figure 2B shows a vascular pedicle (white arrow) in the inferior portion of the lesion rising from left ovarian artery.

In the pelvic cavity there was a solid inhomogeneous mass, with necrotic areas (Figure 2).

The patient was transferred to the gynaecological ward for a suspected adnexal neoplasia.

Further investigation showed high tumour markers with CEA level of 3,3 ng/ml and Ca 19.9 level of 81,2 UI/ml; Ca 125, α-fetoprotein and β-HCG were negative. Serum immunofixation was negative for monoclonal components.

Vaginal ultrasound confirmed the presence of a left ovarian mass; it measured 88 mm. There was no free fluid in the abdominal cavity.

A bone marrow biopsy and aspiration were performed, molecular evaluation showed a polyclonal rearrangement of the IgH gene.

The patient underwent a laparotomic bilateral annessiectomy; operative time length and mean haemoglobin drop value results were 60 min and 125 ml respectively. No adhesions of tumors and other organs, no intra-operative and post-operative complications were observed. Cytological examination of the peritoneal washing was negative for neoplastic cells.

A histopathological examination revealed a high grade B-cell ovarian lymphoma.

**Figure 3:**
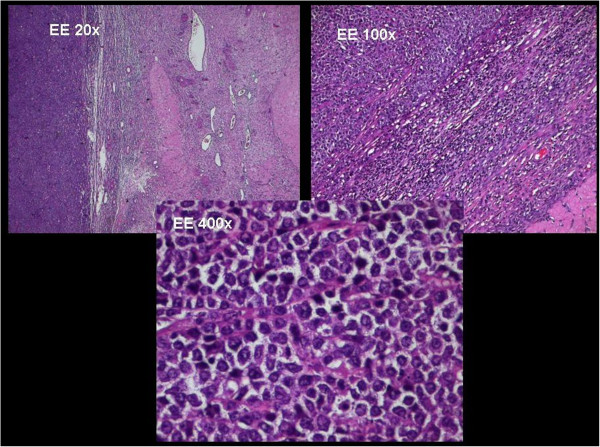
**Histological examination of ovarian lymphoma.** Hematoxylin and eosin-stained sections (original magnifications X20, X100 and X400) show a lymphoid proliferation of small B-cells with a widely monotonous growth pattern, suggestive for high-grade B-cell lymphoma. The round nuclei have finely clumped and dispersed chromatin with multiple basophilic medium size nucleoli, often located in paracentral side.

**Figure 4:**
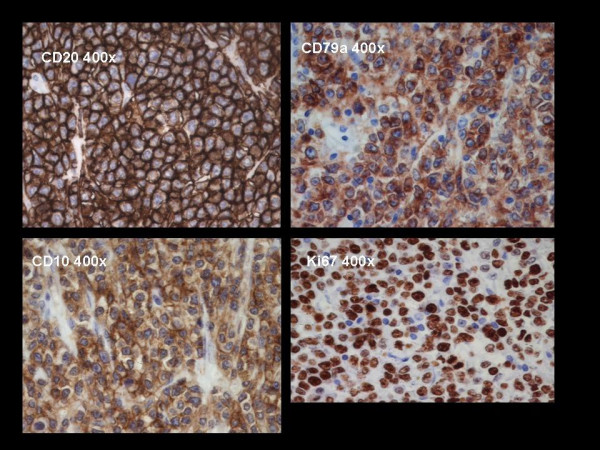
**Immunohistochemical pattern.** The slides show neoplastic B-cell CD20, CD79a (B cell associated antigen) and CD10 positive. The mitotic index of the lymphoma is very high with a Ki67 that is approssimately equal to 100.

Immunohistochemical evaluation revealed the presence of tumour cells that were positive for B-cell markers with a high proliferation rate (CD20, CD10 and CD79a) (Figure 3 and 4).

The FISH test was negative for c-myc t (8;14) translocation and PCR was negative for t (14;18) translocation. These patterns indicated a not translocated Burkitt's lymphoma.

According to Ann Arbor Staging a diagnosis of stage IV A was confirmed. After surgical resection the patient was treated with an intensive G-mall chemotherapy protocol. In older patient (> 55 years) G-m all protocol contemplates a pre-phase of 5 days (cyclophosphamide 200 mg/m^2^ iv and prednisone 60 mg/m^2^ ), cycle A^1^ from day 7, cycle A^2^ from day 49 and a cycle A^3^ from day 98 (rituximab 375 mg/m^2^ iv, dexamethasone 10 mg/m^2^ po , methotrexate 500 mg/m^2^ iv and 12 mg i.th., ifosfamide 400 mg/m^2^ iv, cytarabine 2x60 mg/m^2^ iv, VP16 60 mg/m^2^ iv), cycle B^1^ from day 28, cycle B^2^ from day 77 and cycle B^3^ from day 119 (rituximab 375 mg/m^2^ iv, dexamethasone 10 mg/m^2^ po, vincristine 1mg iv, methotrexate 500 mg/m^2^ iv and 12 mg i.th., ciclofosfamide 200 mg/m^2^ iv, adriamicina 25 mg/m^2^ iv).

Post-operatively and post-CHT CT scan showed a volumetric reduction of the enlarged lymph nodes. Nevertheless the patient decided to sign her discharge papers, despite her poor physical condition and against the advice of her doctors.

## Discussion

Burkitt’s lymphoma (BL) is a highly aggressive, mature B-cell lymphoma that shows an extremely rapid growth rate [1,2]. In 2008, the World Health Organization (WHO) classification recognizes three clinical subtypes of BL: African or endemic, sporadic and immunodeficiency associated. Burkitt’s lymphoma of the ovaries can be primary or more frequently secondary to a systemic neoplastic process [3]. The ovary is often a site of secondary lymphomatous diffusion but less than 10% of ovarian lymphomas have their onset in the ovary [2]. The secondary involvement may be an initial clinical presentation of occult extra-ovarian disease or a manifestation of widely disseminated disease [4].

Primary ovarian lymphoma (POL) is an event that accounts for 0.5% of NHL and 1.5% of all ovarian neoplasia, usually bilateral and often associated with ascites [4-6].

In 1976, POL was defined by Fox and Langley with the following criteria [6]:

(a) the disease has to be confined to the ovary,

(b) absence of disease in the blood and bone marrow,

(c) the extraovarian deposits, if any, should appear at least after few months.

The most frequent clinical symptoms of gynaecologic BL are pelvic pain, abnormal vaginal bleeding, bowel obstruction and abdominal mass with a rapid growth; sometimes it shows unspecified symptoms with a more difficult differential diagnosis [7]. Burkitt’s lymphoma is generally defined as a neoplasia with a typical, but not pathognomonic aspect of starry-sky pattern induced by mitotes of macrophages, with neoplastic cells that tend to form pseudoacini, structured in cords and nests. In our case the clinical presentation of lymphoma did not appear with the classical symptoms (fever, night sweats and weight loss) since the patient referred to the hospital manifesting the occurrence of hypoaestesia and weakness of both the upper limbs. After a MRI evaluation that identified a neurological compression of the cervical nerve roots by multiple lymphoadenopathy, a CT scan was performed and showed an adnexal mass. After surgical removal the histological, molecular and cytogenetic features indicated a not translocated Burkitt’s lymphoma. According to the WHO classification up to 10% of BL cases may lack a MYC translocation, nevertheless MYC has a not specific involvement in BL [8].

Medical studies suggest that the origin of lymphomas in the ovaries is associated with the presence of preexisting lymphoid tissue in the ovary. Nevertheless other authors reveal that reactive lymphocytes may secondarily involve the ovary in response to inflammation (PID, endometriosis) or autoimmune diseases and then they may undergo malignant change and give rise to POL [5,6]. The medical literature suggests that Burkitt’s lymphoma has to be considered in an adult woman with an ovarian mass, excluding granulose tumours , granulocytic sarcoma, poorly differentiated carcinoma, metastasis and epithelial ovarian neoplasia [3]. Being aware of the clinical impact of this potentially curable lymphoma, our case showed the importance of a detailed evaluation and multidisciplinary collaboration.

The prognosis of our patient, according to the International Prognostic Index of non-Hodgkin’s lymphoma, is a 5-year survival of 43%, corresponding to an high-intermediate risk [9]. The International Prognostic Index (IPI) is a clinical tool used to predict the prognosis of patients with aggressive non-Hodgkin's lymphoma. For the evaluation of the prognosis this clinical score considers Ann Arbor staging, age, elevated serum lactate dehydrogenase (LDH), performance status, and number of extranodal sites of disease.

Written informed consent was obtained from the patient for publication of this Case report and any accompanying images. A copy of the written consent is available for review by the Editor-in-Chief of this journal.

The patient of our case gave her consent concerning the case report.

## Competing interests

The authors disclose any commercial interest, any financial interest and any potential conflict of interest of other nature. No financial support has been required for this study.

## Authors’ contributions

PB has conceived and designed the study, has been performed the surgical intervention and has given final approval of the version to be published; FT and GC have supervised the work and were involved in acquisition of data; MM has made CT scan images processing in final form for publication; VG and MV have followed the patient during hospitalization and have drafted the manuscript. All authors have read and approved the final manuscript.
